# Full solid-state magnetic refrigeration device toward thermal management

**DOI:** 10.1073/pnas.2534684123

**Published:** 2026-04-20

**Authors:** Yuan Lin, Victorino Franco, Jing Wang, Jia Yan Law, Yunzhong Chen, Jirong Sun, Tongyun Zhao, Fengxia Hu, Baogen Shen

**Affiliations:** ^a^Beijing National Laboratory for Condensed Matter Physics, Institute of Physics, Chinese Academy of Sciences, Beijing 100190, People’s Republic of China; ^b^School of Physical Sciences, University of Chinese Academy of Sciences, Beijing 101408, People’s Republic of China; ^c^Multidisciplinary Unit for Energy Science, Condensed Matter Physics Department, University of Seville, Seville 41080, Spain; ^d^Songshan Lake Materials Laboratory, Dongguan, Guangdong 523808, People’s Republic of China; ^e^Ganjiang Innovation Academy, Chinese Academy of Sciences, Ganzhou, Jiangxi 341000, People’s Republic of China; ^f^Ningbo Institute of Materials Technology and Engineering, Chinese Academy of Sciences, Ningbo, Zhejiang 315201, People’s Republic of China

**Keywords:** thermal management, full solid-state, magnetic refrigeration device, hybrid regeneration

## Abstract

The demand for thermal management of electronics is rapidly increasing due to the prosperity of information technology. However, the two major alternatives, convective cooling and vapor compression technologies, have limitations for the effective refrigeration of microchips, namely the low convective heat-transfer coefficient of gas, and the need of large compressors, not to mention the low efficiency of compressor cooling and its use of greenhouse refrigerant. Herein, we establish a full solid-state magnetic refrigeration device with scalability and simple structure based on hybrid regeneration utilizing solid heat transfer materials, aiming at offering active point-to-point thermal management through solid-to-solid contact to targets with different footprints. Even with the intrinsic experimental imperfections of a laboratory demonstrator, the device shows a high heat-transfer coefficient *h* of 336 W m^−2^ K^−1^ (typically forced air convection by electric fans have <100 W m^−2^ K^−1^), a high unit cascade heat-transfer coefficient *h*/*n* of 168 W m^−2^ K^−1^, and a large area cooling power *W* of 0.72 W cm^−2^ at the temperature difference between environment *T*_e_ and hot object *T*_o_ of −20 K, which make our full solid-state design the best in the field of thermal management compared to reported full solid-state caloric devices.

With the development of technology and growth of the population, refrigeration has become an indispensable part of modern society. However, the wide use of vapor compression refrigeration places a heavy burden on environment: It utilizes chemical refrigerant which aggregates greenhouse effect, and wastes considerable energy due to the low efficiency. Of particular interest is the thermal management of high-performance electronics, with increasing dissipated power but a smaller footprint of devices. The use of large compressors and the low convective heat-transfer coefficient of gas are hinders to fast remove heat from small-size electronics. Thus, a green, scalable, and effective refrigeration technology is urgently needed. Caloric effect produced upon application/removal of an external driving field is a tangible solution to these problems (1). Among different calorics, like electrocaloric (2–4) and mechanocaloric effects (5, 6), magnetocaloric (MC) refrigeration is closer to large-scale applications (7, 8).

However, traditional passive/active magnetic regeneration (PMR/AMR) models cannot satisfy the demand of electronics thermal management. These systems usually rely on fluids as heat transfer material, which is not compatible with electric circuits (8, 9). Besides, though several full solid-state designs replacing the regenerating fluid by thermal diodes were put forward, it is difficult to practically implement these designs inasmuch as heat leakage of practical k_H_ materials and commercial Peltier elements deviates themselves from ideal thermal diodes, which introduces large energy waste and significantly influences the cooling process (8–10).

For practical purposes, utilizing high thermal conductivity materials (HTCMs) to implement regeneration is a promising alternative to attempts of pursuing an ideal thermal diode, while they still allow the removal of the fluid in the device. Herein, we report a full solid-state MC refrigeration device, based on hybrid regeneration with HTCMs as regenerator toward thermal management of electronics.

## Results and Discussions

**Fig. 1.**
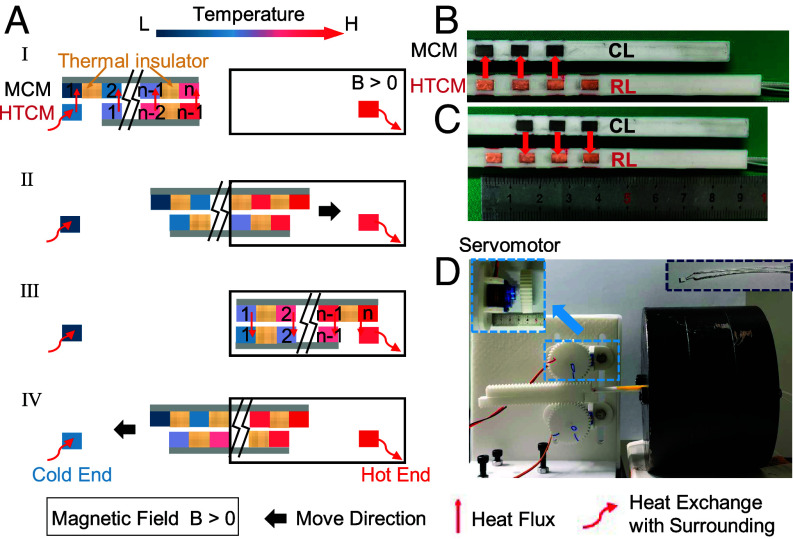
Structure and working principle of the reciprocating full solid-state refrigeration device utilizing HTCMs as regenerators. (*A*) Structure and operating steps of the refrigeration cycle, where I, III show the two static states and II, IV show the two moving states. (*B* and *C*) Photos of the CL and RL at the two static states, where the red arrows denote heat flux. (*D*) Photo of the device where *Insets* show the microservomotor of ~2*2*1 cm^3^ and PT-1000 sensor.

Fig. 1 presents the structure and working principle of the full solid-state MC refrigeration model utilizing HTCMs for regeneration. The core refrigeration unit is composed of two layers which reciprocate to realize refrigeration during the cooling cycle (Fig. 1*A*): a cooling layer (CL) made of magnetocaloric materials (MCMs) and a regeneration layer (RL) made of HTCMs (Fig. 1 *B* and *C*). Each layer of CL or RL contains multiple slices of MCMs or HTCMs, respectively, which are separated by adiabatic materials to block in-layer heat transfer. Heat transfer only exists between the two layers when the MCM slices and HTCM slices become in contact (*SI Appendix*, section SI-1).

During the refrigeration process, there are four states in total (Fig. 1*A*): two static states (I, III in Fig. 1*A*) and two moving states (II, IV in Fig. 1*A*). Following the order from I to IV, each MCM m^th^ slice absorbs heat from the left HTCM (m-1)^th^ slice at static state I and releases heat to the right HTCM m^th^ slice at static state III (*SI Appendix*, section SI-1). Therefore, as the two static states alternate, heat is continuously pumped from the left to the right slice, achieving the aim of refrigeration. Meanwhile, the presented device applies recently reported Hybrid Magnetic Regeneration (HMR) (11) (*SI Appendix*, section SI-2), where a stable temperature gradient forms in both the RL and CL (Fig. 1*A*), which can greatly reduce the heat loss caused by the unstable temperature gradient in the fluid-based AMR/PMR devices (11). Additionally, without fluid and the corresponding pump, such device can be scaled along the width and length, or stacked along the thickness one by one (*SI Appendix*, section SI-1), to offer refrigeration to various target applications with different footprints, ranging from microelectronics to buildings.

**Fig. 2.**
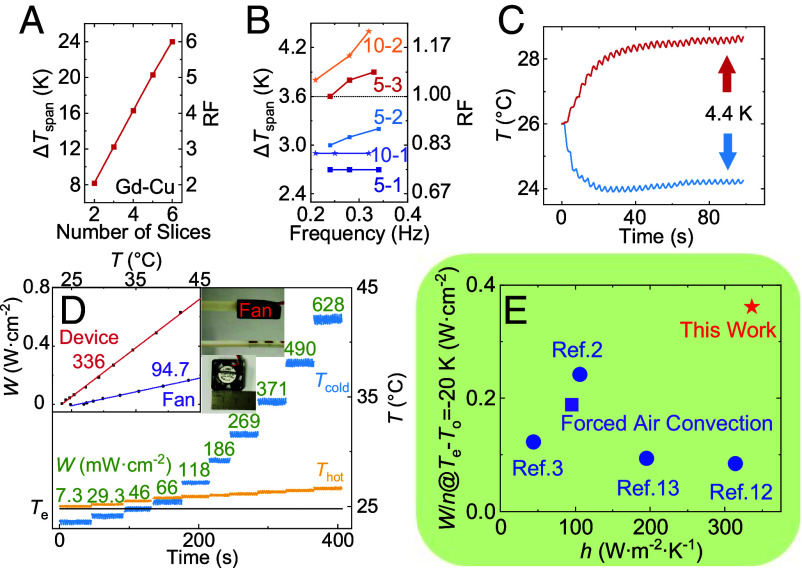
Cooling performance of the full solid-state device. (*A*) Simulated temperature span and RF as a function of the number of MCM slices. (*B*) Experimental temperature span as a function of frequency for different devices where 5 and 10 mm denote the length and 1,2 and 3 denote the number of Gd slices. The dashed line denotes the Δ$T_{\mathrm{adiabatic}}$ of Gd = 3.6 K. (*C*) Experimental time evolution of the temperature of the cold and hot ends with two Gd slices of 3*10*1 mm^3^ at ~0.35 Hz. (*D*) Experimental cold-end and hot-end temperatures of two Gd slices of 3*5*1 mm^3^ with different cooling power at ~0.35 Hz, where *Insets* show the measured heat-transfer coefficient *h* of the device and microfan with nearly the same input of ~0.2 W, the photo of the microfan and the schematic of microfan measurement. (*E*) Comparison of heat-transfer coefficient *h* and the ratio of area cooling power *W*@ T_e_–T_o_ = −20 K to cascade number *n* of our device with other reputed full solid-state devices (refs. 2, 3, 12, and 13) and the forced air convection by electric fans.

The validation of working principle has been twofold. Initially, finite element simulation was carried out, considering the characteristics of Gd as MCMs (adiabatic temperature change ${\Delta T}_{\mathrm{adiabatic}}$ = 4 K) and Cu as HTCMs. Results show that the $\Delta T_{\mathrm{span}}$ linearly increases with the number of MCM slices (n), while the regeneration factor ($RF={\Delta T}_{\mathrm{span}}/\Delta T_{\mathrm{adiabatic}}$) nearly equals n (Fig. 2*A* and *SI Appendix*, section SI-3).

Next, to experimentally prove the feasibility and practicality, a demonstration device was constructed. With the help of a 3D printer, the support of the CL and RL was made with PLA polymer, which eliminates the heat transfer within each layer. Three Gd slices of 3*5*1 mm^3^ and two Cu slices of 3*5*0.5 mm^3^ (plus two additional ones behaving as cold and hot ends) were embedded into the support (Fig. 1 *B* and *C*). PT-1000 temperature sensors (*Right* *Inset* of Fig. 1*D*) were glued to the back of the leftmost and rightmost Cu slices to monitor the temperature evolution of the hot and cold ends. The CL and RL were connected to rack and pinion mechanisms that were driven by two Arduino controlled microservomotors with the size of 2*2*1 cm^3^ (*Left* *Inset* of Fig. 1*D*), providing the reciprocating movement between the two static states (Fig. 1 *B* and *C*). Devices with two and one Gd slices (and correspondingly one and zero Cu slices) were also tested. The experimentally measured $\Delta T_{\mathrm{adiabatic}}$ of a single Gd slice upon reciprocation was 3.6 K. For the complete device, in line with simulations, the temperature span increased with the number of Gd slices ($\Delta T_{3,span}>\Delta T_{2,span}>\Delta T_{1,span}$) (Fig. 2*B*), but influenced by interface thermal resistance and friction due to imperfect surfaces, it was lower than the simulation limit. It is also validated that the cooling power increases with increasing frequency, which reflects on an increasing temperature span. For three Gd slices, the largest temperature span was 3.9 K at ~0.35 Hz (Fig. 2*B*). Due to the limited power of the microservos, frequency could not be increased further.

To preliminarily check the scalability of the device, we tested the performance of double length Gd slices (3*10*1 mm^3^). Similarly, the temperature span increased with slice number ($\Delta T_{2,span}>\Delta T_{1,span}$) and frequency (Fig. 2*B*). Only two slices of this size could fit the constrained field region of our magnet. Since the mass of Gd and thus the cooling power increased, the largest temperature span of 4.4 K was measured with two Gd slices at ~0.35 Hz (Fig. 2 *B* and *C*).

To quantify cooling performances, the hot-end Cu slice was replaced with a larger one of 3*50*1 mm^3^ to promote heat dissipation, and the heat load at cold-end Cu slice was given by a PT-1000 resistor through adjusting current. The measured total input power of the two microservos is 0.2 W. For comparison, the cooling performance of a microfan AD0205LB-K50 (2.5*2.5*0.6 cm^3^) of 0.22 W is measured (*Inset* of Fig. 2*D* and *SI Appendix*, section SI-4). According to

[1] $$P=-h\;A\;(T_{e}-T_{o}),$$

where *P* is the cooling power (in W), *h* is the heat-transfer coefficient (W m^−2^ K^−1^), *A* is the area (m^2^), *T*_e_, *T*_o_ are the temperature of environment and hot object respectively, our demonstrator, even with imperfect surface thermal contact and insufficient heat dissipation, realizes *h* ~ 336 W m^−2^ K^−1^ (area cooling power *W* ~ 0.72 W cm^−2^ at *T*_e_–*T*_o_ = −20 K) (*Inset* of Fig. 2*D*), three times larger than forced air convection of 94.7 W m^−2^ K^−1^ (W ~ 0.19 W cm^−2^) by electric fans with nearly the same input (typically <100 W m^−2^ K^−1^) (14, 15), exceeding all the full solid-state electrocaloric devices (horizontal axis of Fig. 2*E*, detailed in *SI Appendix*, section SI-5). In other words, with our device replacing electric fans to dissipate heat of electronics, the performance of microchips can be tripled, like laptops, portable medical equipment, etc. Besides, due to the larger thermal conductivity of Gd (~10 W m^−1^ K^−1^) compared to PVDF-based polymer (~0.2 W m^−1^ K^−1^) and PbSc_0.5_Ta_0.5_O_3_ (~1.4 W m^−1^ K^−1^), it requires less cascade number *n* to reach a considerable *h* and area cooling power *W*. Therefore, the presented device exhibits an outstanding unit cascade area cooling power *W/n* of 0.36 W cm^−2^ at T_e_-T_o_ = −20 K (*h/n* = 168 W m^−2^ K^−1^), showing the large potential for improvement for the presented device through cascading more slices (vertical axis of Fig. 2*E*).

## Conclusion

A full solid-state MC refrigeration device is constructed by utilizing HTCMs as regenerator, where a recently reported hybrid regeneration mode is applied. Even with the experimental imperfections of a laboratory demonstrator, like friction, surface thermal resistance, and insufficient heat dissipation, a high heat-transfer coefficient of 336 W m^−2^ K^−1^ (*h/n*=168 W m^−2^ K^−1^), an outstanding area cooling power *W* of 0.72 W cm^−2^ (*W/n* = 0.36 W cm^−2^) at T_e_–T_o_ = −20 K, an RF larger than 1, and a temperature span of 4.4 K can be easily realized. The absence of fluid and the related pump endows such device with simple structure and scalability to provide point-to-point active cooling for different-size targets. Our work proves the feasibility and practicality of the full solid-state design and provides a possibility for thermal management. Future perspectives include improvement of the thermal contact between the layer, increased frequency of operation, cascading more slices, and exploration of MCMs with suitable working temperature for thermal management at higher temperatures.

## Materials and Methods

Finite element simulations were conducted by COMSOL Multiphysics 5.6 software. 3D printing was accomplished by a 3D printer CREALITY K1 Max. MCM and HTCM slices were embedded into the PLA support by pressure. The microservomotor used is TIANKONGRC SG90 (~2*2*1 cm^3^). The current of PT-1000 sensor is provided by KEITHLEY 2611B SourceMeter, and the voltage is measured by KEITHLEY 2182A Nanovoltmeter.

## Supplementary Material

Appendix 01 (PDF)

Dataset S01 (XLSX)

Dataset S02 (XLSX)

Dataset S03 (XLSX)

Dataset S04 (XLSX)

Dataset S05 (XLSX)

## Data Availability

Study data are included in the article and/or supporting information.
