# Supplementary material for: Full solid-state magnetic refrigeration device toward thermal management
Source: Proc Natl Acad Sci U S A. 2026 Apr 20;123(17):e2534684123. doi: 10.1073/pnas.2534684123 (PMC13123841; doi:10.1073/pnas.2534684123)
Supplement: Supplementary file 1 — Appendix 01 (PDF) [file pnas.2534684123.sapp.pdf]

Supporting Information for

Full solid-state magnetic refrigeration device toward thermal management

Yuan Lin,[1,2] Victorino Franco,[3]* Jing Wang,[1,2]* Jia Yan Law,[3] Yunzhong Chen,[1,2] Jirong Sun,[1,2,4] Tongyun Zhao,[1,5] Fengxia Hu,[1,2,4]*, and Baogen Shen[1,2,5,6]*

[1]Beijing National Laboratory for Condensed Matter Physics, Institute of Physics, Chinese Academy of Sciences, Beijing, 100190, P. R. China.
[2]School of Physical Sciences, University of Chinese Academy of Sciences, Beijing, 101408, P. R. China.
[3]Multidisciplinary Unit for Energy Science, Condensed Matter Physics Department, ICMS-CSIC, University of Seville, Seville, 41080, Spain
[4]Songshan Lake Materials Laboratory, Dongguan, Guangdong, 523808, P. R. China.
[5]Ganjiang Innovation Academy, Chinese Academy of Sciences, Ganzhou, Jiangxi, 341000, P. R. China.
[6]Ningbo Institute of Materials Technology & Engineering, Chinese Academy of Sciences, Ningbo, Zhejiang, 315201, China

* Victorino Franco, Jing Wang, Fengxia Hu, Baogen Shen.
Email: vfranco@us.es (V. F.); wangjing@iphy.ac.cn (J. W.); fxhu@iphy.ac.cn (F. H.); shenbg@iphy.ac.cn (B. S.)

This PDF file includes:

Supporting text 1-5
SI References

Supporting Information(SI)-Extended Methods

SI-1. Details of the device design

During the refrigeration process, there are four states in total (Fig.1A): two static states (I,III in Fig.1A) and two moving states (II,IV in Fig.1A). The detailed working process is described below:

(I)/(III): Both the RL and the CL are (out of)/(inside) the magnetic field and keep static for thermal exchange and stabilization. The MCM slices did experience a temperature (decrease)/(increase) when they were (extracted from)/(inserted into) the magnetic field region before. Each MCM slice contacts with the (left)/(right) HTCM slice: the mth MCM slice contacts with the $(m-1)^{th}$/(m^{th}) HTCM slice and (absorbs)/(releases) heat (from)/(to) it (I,III in Fig.1A).

(II)/(IV): Both the RL and the CL move (into)/(out of) the magnetic field. During this process, the MCM slices experience an (increase)/(decrease) in temperature. The CL moves faster than the RL, so each MCM slice separates from the (left)/(right) HTCM slice before (entering)/(leaving) the field: the mth MCM slice leaves the $(m-1)^{th}$/(m^{th}) HTCM slice and approaches the (m^{th})/$(m-1)^{th}$ HTCM (II,IV in Fig.1A).

Following the order from I to IV, each MCM (m^{th}) slice absorbs heat from the left HTCM $(m-1)^{th}$ slice at static state I and releases heat to the right HTCM m^{th} slice at static state III. Therefore, as the two static states alternate, heat is continuously pumped from the left to the right slice, achieving the aim of refrigeration.

The length of the adiabatic materials should be longer than that of the slices. The length of the MCM slices and HTCM slices could be different. To maintain the stable temperature gradient formed in the MCM and HTCM slices, the length of the adiabatic materials in the regeneration layer (RL) should be longer than that of MCM slices, and the length of the adiabatic materials in the cooling layer (CL) should be longer than that of HTCM slices. If the length of the adiabatic materials in the regeneration layer (RL) is shorter than that of MCM slices and the length of the adiabatic materials in the cooling layer (CL) is shorter than that of HTCM slices, during the movement (II,IV in Fig.1A), each MCM slice will contact two adjacent HTCM slices at the same time and each HTCM slice will contact two adjacent MCM slices at the same time. As a result, the heat will be pumped reversely from the hot end to the cold end, introducing regeneration losses and thus lowering the cooling performances.

A method for enhancing performance is to stack different elements along the thickness one over the other. This does not simply mean putting several independent devices together along the thickness. Actually, this full solid-state device can contain several regeneration layers (RL) made of HTCM slices and cooling layers (CL) made of MCM slices at the same time. Each RL should be sandwiched by two CLs, and each CL should be sandwiched by two RLs, i.e., a device can contain k CLs and (k±0,1) RLs. One RL and one CL are the simplest configuration. For devices with more than one RL and one CL, the working process and working principle are the same as the simplest one introduced in the manuscript. The only difference is that at the static states I and III (Fig.1A), each RL exchanges heat with the two adjacent CLs, and each CL exchanges heat with two adjacent RLs, which can boost the heat exchange rate, further improving cooling performances.

SI-2. Difference between hybrid magnetic regeneration and passive/active magnetic regeneration

The aim of regeneration is to realize a temperature span larger than the adiabatic temperature change of magnetocaloric materials. The core idea is to establish a stable temperature gradient between the cold end and the hot end of the device. Usually, magnetocaloric refrigeration devices are composed of magnetocaloric materials and heat-transfer materials. If the stable temperature gradient forms along the heat-transfer materials, it is called passive magnetic regeneration (PMR) (see Fig.1A of Ref.1); if the stable temperature gradient forms along the magnetocaloric materials, it is called active magnetic regeneration (AMR) (see Fig.1B of Ref.1); here, the stable temperature gradient forms along both the magnetocaloric materials and the heat-transfer materials (high thermal conductivity materials), the manifestation of which is markedly different from PMR/AMR, so we call it hybrid magnetic regeneration (HMR) (see Fig.1C of Ref.1).

For passive magnetic regeneration (PMR), the heat-transfer fluid is static while the magnetocaloric material (MCM) moves between the cold and the hot ends to establish a stable

temperature gradient in the heat-transfer fluid with the cooperation of magnetic field (Fig.1A of Ref.1) (Ref.2). The volume ratio of MCMs in PMR is so low that the cooling power is limited.

For active magnetic regeneration (AMR), the MCM separated into slices is static while the heat-transfer fluid moves between the cold and hot ends to establish a stable temperature gradient in the MCM with the cooperation of magnetic field (Fig.1B of Ref.1). The volume ratio of MCMs in the whole device increases, thus effectively improving the cooling power, overcoming the main weakness of PMR (Ref.3). However, the cooling performance of AMR has yet to satisfy daily application. There is temperature nonuniformity in the heat-transfer fluid, which continuously causes heat losses during the whole refrigeration cycle; Worse still, since not all the fluid is in sufficient contact with MCMs at a certain time, there is always inert part of fluid in the cycle, which restricts the regeneration efficiency and thus cooling performances.

For hybrid magnetic regeneration (HMR), the role of MCMs and heat-transfer materials is nearly identical. Both MCMs and heat-transfer materials (using high thermal conductivity materials, HTCMs) are divided into independent slices (Fig.1C of Ref.1). With the cooperation of magnetic field, MCM and heat-transfer material slices perform relative motion. As a result, a stable temperature gradient forms in both the MCM and heat-transfer material. For HMR, the high volume ratio of MCMs in the device is kept, thus inheriting the high cooling power advantage of AMR. Besides, with stable temperature gradient forming in both MCMs and heat-transfer materials, the regeneration loss caused by unwanted heat transfer due to unstable temperature gradient is eradicated. Furthermore, at any time of the cycle, each HTCM slice is in sufficient contact with a MCM slice, effectively lowering inert part. As a result, heat can be pumped more efficiently. Regeneration efficiency and thus cooling performance in HMR could be further increased. On the other hand, full solid-state design abandons fluid and pump, which simplifies the device structure and provides the device with scalability to face different situations. Also, possible high moving speed of solid and high thermal conductivity of HTCMs offer opportunities to further improve working frequency and thus cooling power (Ref.1).

To conclude, in PMR/AMR, the stable temperature gradient forms in either the heat-transfer materials or MCMs respectively, while in HMR, the stable temperature gradient forms simultaneously in the heat-transfer materials and MCMs. Inheriting the high MCM volume ratio of AMR, HMR could also overcome the main weakness of PMR. Compared to AMR, the stable temperature gradient reduces the heat loss caused by unstable temperature gradient in heat-transfer materials, and the inert ratio of heat-transfer materials is lowered, resulting in an improved regeneration efficiency and refrigeration performances. Additionally, the full solid-state design simplifies the device structure and endows the device with scalability to provide refrigeration for targets of different sizes. Also, possible high moving speed of solid and high thermal conductivity of HTCMs offer opportunities to further improve working frequency and thus cooling power.

SI-3. Details of finite element simulations (Dataset02, Dataset03)

The finite element simulations were carried out through COMSOL Multiphysics 5.6 software (Ref.1).In simulations, the width and length of both the MCM and HTCM slices are set as 3 mm and 5 mm respectively, and the thickness of MCM and HTCM slices is set as 1 mm and 0.5 mm respectively. Gd and Cu are selected as the MCM and HTCM, respectively. The adiabatic temperature change of Gd is set as 4 K, which means its temperature increases instantly by 4 K when entering the magnetic field, and decreases instantly by 4 K when leaving the magnetic field. Heat transfer only exists between MCM and HTCM slices when they contact each other. Other surfaces are set as adiabatic boundaries. The cooling layer composed of several MCM slices and the regeneration layer composed of several HTCM slices move, as indicated in the manuscript, to realize refrigeration. The temperature in the same slice is not homogeneous, which means there is heat transfer inside each slice. The numerical model of the device can be written as a three-dimension heat diffusion equation,

$$\rho C_p \frac{\partial T}{\partial t} + \nabla \cdot \boldsymbol{q} = Q$$

$$\boldsymbol{q} = -k\nabla T$$

where ρ (in unit of $kg \cdot m^{-3}$), C_p (in unit of $J \cdot kg^{-1} \cdot K^{-1}$), and k (in unit of $W \cdot m^{-1} \cdot K^{-1}$) are density, specific heat at constant pressure, and thermal conductivity of specific materials. T (in unit of K), Q (in unit

of W·m^{-3}) are temperature, and the heat produced by magnetocaloric effect respectively. The heat transfer is continuous and conservative.

Firstly, we employed different types of magnetocaloric materials (MCM) including Gd and $La(Fe_{0.92}Co_{0.08})_{11.7}Si_{1.3}$ (LFS) and high thermal conductivity materials (HTCM) including Cu, Ag and aluminum-diamond alloy (ALC) in the simulation, where the thickness of cooling layer is set as 1 mm and that of regeneration layer is set as 0.5 mm. The length and width of all the slices are set as 5 mm and 3 mm respectively. The adiabatic temperature change of both Gd and LFS is set as 4 K. The related parameters of these materials are shown in Dataset02 (Ref.1,7-10). As shown in Dataset03, the temperature span increases linearly with the number of slices, and the regeneration factor nearly equals the number of slices for all the combinations of MCMs and HTCMs.

Secondly, we fix the type of MCM and HTCM as Gd and Cu, the length and width of all the slices as 5 mm and 3 mm respectively, but change the thickness of the cooling layer and regeneration layer. As shown in Dataset03, the temperature span increases linearly with the number of slices, and the regeneration factor nearly equals the number of slices for all the combinations of different thickness of MCMs and HTCMs.

Thirdly, we fix the type of MCM and HTCM as Gd and Cu, the thickness of the cooling layer and regeneration layer as 1 mm and 0.5 mm respectively, but change the length and width of all the slices. As shown in Dataset03, the temperature span increases linearly with the number of slices, and the regeneration factor nearly equals the number of slices for all the combinations of different width and length.

To conclude, in finite element simulation, we aim to study the relation between the cooling performances and the design of device structure, so we fix the adiabatic temperature change $\Delta T_{adiabatic}$ of Gd, LFS as 4 K, and ignore interface thermal resistance and friction. In this ideal situation, the temperature span increases linearly with number of slices and the regeneration factor nearly equals the number of slices. Unlike traditional passive/active regeneration devices whose temperature span is not easy to tell from the device structure, even in the ideal situation (Ref.4-6), this direct relation in our full solid-state hybrid magnetic regeneration device could simplify the design of devices. The presented device with this direct relation and simple structure holds high potential to be practically applied.

SI-4. Measurement of forced convective heat-transfer coefficients of electric fans (Dataset04)

In our device, the nominal power of the employed micro-servo TIANKONGRC SG90 (~ 2*2*1 cm^3) is ~ 0.25 W at 5 V, so the total input of our device is no larger than 0.5 W (two micro-servos are employed in the device). For comparison, the convective heat-transfer coefficient of a micro-fan AD0205LB-K50 (2.5*2.5*0.6cm^3) with the same nominal power of 0.5 W is measured, which is specifically designed to dissipate heat for laptops and chips. To further dispel the concern, we directly measured the actual input of the micro-servos and micro-fan while in operation. The measured total input power drawn by the two micro-servos during the cooling cycle is 0.2 W at 5 V, while for the micro-fan, the measured actual input is 0.22 W at 5 V, which are nearly the same.

The micro-fan AD0205LB-K50 (2.5*2.5*0.6cm^3) is shown in the inset of Fig.2D. To measure the heat-transfer coefficient of the micro-fan, the regeneration layer keeps static, and the micro-fan is fixed on top of the regeneration layer (See the inset of Fig.2D). The leftmost Cu slice, i.e., the cold-end Cu slice is heated by the PT-1000 resistance attached to the back of the slice while the electric fan helps to dissipate the generated heat. The temperature of the Cu slice is also monitored by the PT-1000 resistance. Through changing the current and thus the heating power, the area cooling power at different temperature differences is measured (See Dataset04). The slope of the fitting line is the forced air convective heat-transfer coefficient *h* of the micro-fan. The coefficient *h* is related to the distance between the object and the micro-fan, so we measured the coefficient *h* at different distances. As shown in Dataset04, the *h* of the micro-fan for 0.22 W power input is 94.7 W·m^{-2}·K^{-1}@ 0 cm, 87.1 W·m^{-2}·K^{-1}@ 0.5 cm, 69.2 W·m^{-2}·K^{-1}@ 1 cm, indicating the closer the fan, the larger the *h*, in line with the expectation (0 cm, 0.5 cm, 1 cm denote the distance between the regeneration layer and the micro-fan shown in the inset of Fig.2D). We further measured the coefficient *h* of a powerful fan (Φ 8.5*2.5 cm^3) with a nominal power of 5 W, which is 99.4 W·m^{-2}·K^{-1}@ 0 cm (See Dataset04), fitting well with the statement in references (Ref.11-13) that the coefficient of forced air convection is usually lower than 100 W·m^{-2}·K^{-1}. For larger objects in

constrained space, like CPU in computer case, the coefficient *h* of forced convection would be further lowered.

To conclude, with the same input power of ~0.2 W, our device shows a *h* of 336 $W \cdot m^{-2} \cdot K^{-1}$, about 3.5 times larger than the forced air convection by electric fans of 94.7 $W \cdot m^{-2} \cdot K^{-1}$, which proves the significant application potential in thermal management of our device. In practical applications, the coefficient of forced air convection would be smaller for larger objects in constrained space.

SI-5. Details of the comparison shown in Figure 2E (Dataset05)

Analogously to forced convection, the relation between area cooling power of the full solid-state device and the temperature of the object is simplified as Equation (1):

$$P = -h \cdot A \cdot (T_e - T_o) \qquad (1)$$

where *P* is the cooling power (in unit of W), *h* is the heat-transfer coefficient ($W \cdot m^{-2} \cdot K^{-1}$), *A* is the area ($m^2$), T_e, T_o are the temperature of environment (heat sink) and hot object (heat source), respectively (more precisely, for refrigeration devices, T_e should be the temperature realized at zero load rather than ambient temperature).

Heat-transfer coefficient *h* and unit cascade heat-transfer coefficient *h/n* (h/cascading number *n*) are the intrinsic properties of the device. Due to the lack of high-temperature experiment data in the referenced literatures, we extract the *h* and thus *h/n* of the referenced electrocaloric devices from the experiment around room temperature. More specifically, *h* is calculated as $h = W_0/\Delta T_{span,0}$, where W_0 denotes the area cooling power W@T_e-T_o = 0 K, and $\Delta T_{span,0}$ denotes the ΔT_{span} at zero load.

(1) For the electrocaloric device in Ref.14 (n = 1) (Fig.2 of Ref.14), at 66.7 $MV \cdot m^{-1}$, ΔT_{span} at zero load is 2.8 K (Fig.S7B of Ref.14), and the W@T_e-T_o = 0 K is 29.7 $mW \cdot cm^{-2}$ (Fig.3E of Ref.14), both of which are clearly stated in Ref.14.

(2) For that in Ref.15 (n = 1) (Fig.1d of Ref.15), ΔT_{span} at zero load is approximately 8 K (Fig.3d of Ref.15), and according to the reported mass of 16.96 mg and electrode diameter of 20 mm, W@T_e-T_o = 0 K is 35 $mW \cdot cm^{-2}$ related to 6.5 $W \cdot g^{-1}$ (Fig.3d of Ref.15), both of which are clearly stated in Ref.15.

(3) For that in Ref.16 (n = 9) (Fig.3 of Ref.16), ΔT_{span} at zero load is 4.3 K (read from Fig.4D of Ref.16), and the W@T_e-T_o = 0 K related to 85 mW is 135 $mW \cdot cm^{-2}$ (Fig.4D of Ref.16), which is clearly stated in Ref.16.

(4) For that in Ref.17 (n = 6) (Fig.1 of Ref.17), at 80 $MV \cdot m^{-1}$, ΔT_{span} at zero load is 8.8 K (Fig.4C of Ref.17), and the W@T_e-T_o = 0 K is 172 $mW \cdot cm^{-2}$ (Fig.4C of Ref.17), both of which are also clearly stated in Ref.17.

Only the 4.3 K for Ref.16 is read from Fig.4D of Ref.16, while all the other data are clearly reported in the text of related references.

According to these W@T_e-T_o = 0 K and ΔT_{span} at zero load, the *h* is calculated: 106, 44, 314, 195 $W \cdot m^{-2} \cdot K^{-1}$ for Ref.14, 15, 16, 17 respectively. Then, with the cascade number *n*, unit cascade heat-transfer coefficient *h*/*n* can be calculated: 106(n=1), 44(n=1), 35(n=9), 33(n=6) $W \cdot m^{-2} \cdot K^{-1}$ for Ref.14, 15, 16, 17 respectively.

Next, the area cooling power *W* and unit cascade area cooling power *W*/*n* at specific temperature span (T_e-T_o = -20 K) can be derived according to Equation 1. The W@ T_e-T_o = -20 K is 242, 123, 763, 563 $mW \cdot cm^{-2}$ for Ref.14, 15, 16, 17 respectively, and the W/n@ T_e-T_o = -20 K is 242, 123, 85, 94 $mW \cdot cm^{-2}$ respectively. All of these data are included in Dataset05.

Finally, the comparison of these four parameters *h*, *h*/*n*, *W*, *W*/*n* among the presented device, forced air convection by electric fans, and the reputed electrocaloric devices demonstrates the excellent thermal management performance of our presented device.

SI References

1. Y. Lin *et al.*, A full solid-state conceptual magnetocaloric refrigerator based on hybrid regeneration. *Innovation* **5**, 100645-100645 (2024).
2. G. V. Brown, Magnetic heat pumping near room-temperature. *J. Appl. Phys.* **47**, 3673-3680 (1976).
3. J. Barclay, W. Steyert. Active magnetic regenerator. U.S. Patent 4,332,135 (1982).
4. S. Qian *et al.*, High-performance multimode elastocaloric cooling system. *Science* **380**, 722-727 (2023).
5. J. Li *et al.*, High cooling performance in a double-loop electrocaloric heat pump. *Science* **382**, 801-805 (2023).
6. J. Tusek, A. Kitanovski, U. Tomc, C. Favero, A. Poredos, Experimental comparison of multi-layered La-Fe-Co Si and single-layered Gd active magnetic regenerators for use in a room temperature magnetic refrigerator. *Int. J. Refrig.* **37**, 117-126 (2014).
7. V. K. Pecharsky, K. A. Gschneidner, Magnetocaloric effect from indirect measurements: Magnetization and heat capacity. *J. Appl. Phys.* **86**, 565-575 (1999).
8. K. Fukamichi, A. Fujita, S. Fujieda, Large magnetocaloric effects and thermal transport properties of La(FeSi)(13) and their hydrides. *J. Alloys Compd.* **408**, 307-312 (2006).
9. V. Basso, C. P. Sasso, M. Kuepferling, A Peltier cells differential calorimeter with kinetic correction for the measurement of c(p)(H, T) and Delta s(H, T) of magnetocaloric materials. *Rev. Sci. Instrum.* **81**, 113904 (2010).
10. M. Katter, V. Zellmann, G. W. Reppel, K. Uestuener, Magnetocaloric Properties of La(Fe, Co, Si)(13) Bulk Material Prepared by Powder Metallurgy. *IEEE Trans. Magn.* **44**, 3044-3047 (2008).
11. J. H. Whitelaw, Convective Heat Transfer. *Thermopedia*, 10.1615/AtoZ.c.convective_heat_transfer (2011).
12. P. S. Ghahfarokhi *et al.* (2017) Determination of Forced Convection Coefficient Over a Flat Side of Coil. in *58th IEEE International Scientific Conference on Power and Electrical Engineering of Riga-Technical-University (RTUCON)*, 10.1109/RTUCON.2017.8124759 (2017).
13. M. G. Emmel, M. O. Abadie, N. Mendes, New external convective heat transfer coefficient correlations for isolated low-rise buildings. *Energy and Buildings* **39**, 335-342 (2007).
14. R. Ma *et al.*, Highly efficient electrocaloric cooling with electrostatic actuation. *Science* **357**, 1130-1134 (2017).
15. D. Han *et al.*, Self-oscillating polymeric refrigerator with high energy efficiency. *Nature* **629**, 1041–1046 (2024).
16. Y. Wang *et al.*, A high-performance solid-state electrocaloric cooling system. *Science* **370**, 129-133 (2020).
17. H. Wu *et al.*, A self-regenerative heat pump based on a dual-functional relaxor ferroelectric polymer. *Science* **386**, 546-551 (2024).
